# Antenatal hepatitis B virus sero-prevalence, risk factors, pregnancy outcomes and vertical transmission rate within 24 months after birth in a high HIV prevalence setting

**DOI:** 10.1186/s12879-023-08523-2

**Published:** 2023-10-27

**Authors:** Kerina Duri, Privilege Tendai Munjoma, Hope Mataramvura, Arthur John Mazhandu, Panashe Chandiwana, Tarisai Marere, Felicity Zvanyadza Gumbo, Lovemore Ronald Mazengera

**Affiliations:** 1 Immunology Unit, Faculty of Medicine and Health Sciences (UZ-FMHS), University of Zimbabwe, P.O. Box A178, Avondale, Harare, Zimbabwe; 2 Obstetrics and Gynecological Unit, UZ-FMHS, Harare, Zimbabwe; 3 Paediatric and Child Health Unit, UZ-FMHS, Harare, Zimbabwe

**Keywords:** Antenatal hepatitis B surface antigen sero-prevalence, Risk factors for HBsAg seropositivity in pregnancy, Burden of antenatal HBV/HIV coinfections, Vertical and child-horizontal transmission of HBV

## Abstract

**Background:**

Despite the availability of an effective vaccine, chronic hepatitis B virus (HBV) infections remain a major cause of liver cirrhosis and hepatocellular carcinoma. HBV burden in pregnancy, risk factors and the timing of mother to child transmission remain poorly described especially during this era of lifelong use of Tenofovir/Lamivudine/Efavirenz as firstline for HIV treatment. We aimed to determine the burden of HBV in pregnancy and infants receiving their first dose of HBV vaccine 6 weeks after birth in a high HIV-prevalence setting.

**Methods:**

Pregnant women ≥ 20 weeks’ gestational age were enrolled and followed up as mother-infant dyads from delivery, 6, 24 and 96 weeks after birth. HBV surface antigen (HBsAg) was tested (fresh plasma, immunochromatography) in pregnancy. Women testing HBsAg-seropositive were further evaluated for other four HBV-biomarkers. Maternally HBV exposed babies were tested for HBsAg from birth and HBs-antibodies from 6 months of age. Maternal-infant factors were tested in univariable and multivariable analyses for predictors of HBsAg-seropositivity.

**Results:**

Six hundred HIV-uninfected and 608 HIV-infected women on Tenofovir/Lamivudine/Efavirenz-regimen with median (interquartile range) 350: (87–1477) days of therapy use were enrolled. The overall HBsAg-seroprevalence was 32/1208: 2.65%, 95% confidence interval (CI) [1.74, 3.55]; being 7/600: 1.17%, 95% CI [0.37, 1.97] and 25/608: 4.11%, 95% CI [2.52, 5.68] in HBsAg-monoinfected and HBsAg/HIV-coinfected respectively, disproportionately detected in 31/32: 96.9%, 95% CI [90.8, 100] women presumably HBV-unvaccinated in infancy.

HBV exposed babies tended to be born prematurely (< 37 weeks); 15.2% versus 9.9% in the HBV-unexposed, *p* = 0.009.

In multivariate logistic regression-models with variable elimination, HIV-infection and reported tooth extractions predicted antenatal HBsAg-seropositivity; odds ratios (CI): 3.85 (1.61–10.7) and 2.46 (1.07–5.34), respectively.

None of the exposed infants were HBsAg-seropositive neither before nor after 6 weeks of age. No HBs-antibodies were detected in 23.3% of HBsAg-exposed infants at two years despite having successfully completed the HBV vaccination schedule.

**Conclusion:**

Low and moderate HBV endemics were observed in HIV-uninfected and HIV-infected pregnant women, respectively. This underscores the need to routinely screen for HBV in pregnancy, especially the HIV-infected attending antenatal-care. Being HIV-infected and reported tooth extractions were independent risk factors for maternal HBsAg-seropositivity. Vertical and child horizontal transmissions were both absent, probably due to ~ the 50% frequency of antenatal anti-HBe-antibodies observed. Of concern was the absence of anti-HBs-antibodies in 23.3% of fully vaccinated/maternally HBV-exposed infants by two years. Absence of molecular diagnosis may have underestimated HBV burden.

**Trial registration:**

www.clinicaltrials.gov, trial registration number: NCT04087239.

## Introduction

Hepatitis B virus (HBV) is a carcinogenic DNA virus that causes acute and chronic liver diseases, disproportionately affecting young African adults [1]. Chronic HBV infection is associated with a 15%-40% increased risk of the development of cirrhosis, hepatic failure and hepatocellular carcinoma (HCC) [2]. Such persistent viral infections are characterized by host weak immune responses with subsequent development of ineffective CD8 + T-lymphocyte responses [3, 4], a situation exacerbated by HIV infection endemic in sub-Saharan Africa (SSA). HIV accelerates progression to cirrhosis and liver cancer. On the other hand, HBV infection has been associated with poor CD4 + T-lymphocyte cell recovery despite successful HIV-RNA suppression [5, 6]. Understanding HBV/HIV dynamics in expecting mothers is essential since pregnancy as a condition is characterised by anti-inflammatory Th2-cytokines associated with increased risk of infections.

High sero-prevalence rates of hepatitis B surface antigen (HBsAg) in SSA of up to 20% in pregnant women [7, 8] remain a risk factor for onward vertical transmission. In these settings, HBV is commonly spread from an infected mother to her child at birth or by the horizontal route through mastication of foods, use of unsterile sharps or from an infected sibling to an uninfected child during the first 5 years of life [9]. However, exactly how and when African infants get infected remain poorly described. Elucidating this is critical since HBV infection acquired later in adulthood causes chronic hepatitis in only < 5% of cases, whereas infection in infancy and/or early childhood leads to chronic hepatitis in 95% of cases [10, 11]. This observation underscores the need to strengthen early-life HBV prevention strategies. Maternal screening programs are aimed at identifying the presence of HBsAg. Once the HBsAg-seropositive mothers are identified their exposed babies should receive passive immunoprophylaxis of specific hyperimmune immunoglobulin or monoclonal antibody preparations at birth to reduce the risk of vertical HBV transmission. Active immunoprophylaxis involves the administration of HBV vaccine birth dose followed by 2 doses given at least 4 weeks apart to complete the vaccination series for protection against HBV infections [11].

Universal HBV infant immunisation was instituted in Zimbabwe in 1988 followed by universal access since 1996. The vaccine is combined with the other four antigen preparations to form a pentavalent administered at 6, 10 and 14 weeks after birth. The Zimbabwe Demographic and Health Survey that assesses demography and health information of common infections through sampling a representative sample of ≥11,000 households nationwide every five years since 1988, reported the HBV vaccination coverage of 90% and 83% for the first and third dose, respectively [12]. However, the current administration of first dose of the HBV vaccine at 6 weeks of age rather than at birth remains a great concern as infants are thought to be vulnerable from birth until about 6 weeks of age, yet there is not much local evidence on the burden of perinatal transmission of HBV. Furthermore, there are research gaps in immunoprophylaxis failure rates of fully vaccinated maternally HBV-exposed babies.

The last comprehensive Zimbabwean report on the burden of HBV in pregnancy was described over 20 years ago before the advent of life-long antiretroviral therapy (ART) when HBsAg-seropositivity was 25% in the 1990s [13]. Around 2009, HBV sero-prevalence rates fell to ~ 3.3%, 3.0% and 3.7% in rural, peri-urban and urban pregnant women, respectively [14]. Thus, there is a paucity of contemporary and comprehensive data on HBV-infection in pregnant women and their infants including associated risk factors. Therefore, we aimed to determine the prevalence of HBsAg sero-status in pregnancy, the vertical and horizontal transmission rates in maternally exposed infants receiving their first dose of HBV vaccine at 6 weeks after birth.

We aimed to;

1. Determine HBsAg sero-prevalence in pregnant women, and describe the associated risk factors.
2. Characterise other HBV biomarkers; anti-HBs antibodies, HBeAg, anti-HBe antibodies, anti-HBc-antibodies in all HBsAg sero-positives. Women testing positive for just HBsAg marker only at baseline were re-tested every 6 months.
3. Determine any associations between HBsAg seropositivity and adverse pregnancy outcomes (low birth weight; <2500g, prematurity; < 37 weeks).
4. Determine HBsAg seropositivity rate in maternally HBV exposed babies from birth and 6 weeks of age (vertical transmission) and horizontal transmission from 6 months of age, i.e. at least 2 months following receipt of the last dose of HBV vaccine up until 2 years of age.
5. Determine the immunoprophylaxis failure rate in maternally HBV-exposed but fully vaccinated babies from at least 6 months of age.

## Methods

### Design of University of Zimbabwe birth cohort study (UZBCS)

The University of Zimbabwe College of Health Sciences Birth Cohort study (UZBCS) is a prospective observational cohort of HIV-infected and HIV-uninfected pregnant women in a resource limited setting of high-density suburbs west of Harare, Zimbabwe. Kuwadzana, Dzivaresekwa (Rujeko), Glenview and Budiriro Harare Municipal polyclinics were selected based on higher volumes of maternal and child health services, frequency of HIV seropositivity, and lack of competing research activities targeting the same study population [15]. The UZBCS aims to investigate the role of maternal comorbidities, including co-infections with persistent viruses such as HIV, cytomegalovirus, HBV, and other infectious diseases like syphilis. Non-communicable diseases include maternal nutritional status, hypertensive disorders and their roles on pregnancy outcomes, infant mortality and general health [15]. By design at baseline approximately 50% of the expecting women in the UZBCS were HIV-infected as previously described [15]. In this cohort, lack of plasma HIV-RNA suppression is common within the HIV-infected subgroup mainly due to new HIV diagnosis, hence not being on ART and/or suboptimal duration of ART-exposure [16]. HIV-MTCT rate is low as previously reported [17].

### Inclusion and exclusion criteria

Inclusion criteria included pregnant women ≥ 15 years of age, ≥ 20 weeks of gestation at enrolment, and planning to deliver at any of the selected 4 study sites.

Exclusion criteria was the presence of severe mental health disorders, rendering the women incapacitated to provide informed consent or comply with study procedures as assessed by the study Clinicians as previously described [15].

### Study procedures

The study midwife administered a structured questionnaire to all pregnant women at enrolment aiming at a comprehensive clinical and socio-demographic characterisation. Socio-economic information assessed comprised of employment status, family monthly income, money set aside for food every month, general household characteristics and food security. Further questions addressing maternal life style such as alcohol use, concurrent medications, presence of comorbidities and sexually transmitted infection (STIs), awareness of spouse/intimate partner’s HIV status, including any missed ART doses were asked. At enrolment, the study midwife also performed a full maternal physical examination, including anthropometry of body mass index (BMI) and mid upper arm circumference (MUAC) to assess nutritional status as previously described [15].

### Blood collection and analysis

Ten milliliters of maternal venous blood were collected in EDTA and plain tubes at enrolment. Maternal HIV diagnosis was done from an aliquot of whole blood EDTA using qualitative rapid immunochromatographic assays, SD Bioline HIV-1/2 3.0 (Standard Diagnostics Inc., Kyonggi-do, South Korea) and Abbott’s Determine*®* HIV*-*1/2*.* Western Blot was the tie breaker for any indeterminate test results as previously described [15].

Absolute CD4^+^ T-lymphocyte counts in whole blood EDTA samples were enumerated within a maximum of 6 h after sample acquisition using a Partec Cyflow counter (Cyflow, Partec, Munster, Germany) [15].

Plasma was processed for HIV RNA load testing and stored at -80 °C as previously described [15].

Assessment of serum liver function enzymes alkaline phosphatase (ALP), alanine transaminase (ALT), aspartate transaminase (AST), gamma-glutamyl transferase (GGT) including bilirubin was done using Beckman Coulter AU680 chemistry analyser (Krefeld, Germany) as previously described [15].

### Testing of maternal HBsAg

Plasma from all the pregnant women were screened for HBsAg using immunochromatographic SD Bioline HBsAg WB test, Gyeonggi-do, Korea at concentration between 0.52–2.06 IU/ml in the World Health Organisation (WHO) International Biological Reference Preparation for HBsAg [03/262]. All samples were confirmed on a colloidal gold enhanced immunochromatographic assay Advanced Quality ™ one step HBsAg test with a lower detection limit of 1 ng/ml at 15 min as per the manufacturers’ instructions. The SD Bioline HBsAg test kit has a sensitivity and specificity of 100% and 99%, respectively as per the WHO evaluation. In addition, this kit was approved by the Ministry of Health and Child Care for diagnostic use in Zimbabwe. The One Step Advanced Quality HBsAg kit is ISO 13485 certified, and has a 100% sensitivity and a 99.4% specificity.

### Testing of other HBV biomarkers in HBsAg seropositive women and infants

All women confirmed for the presence of HBsAg were further tested for the presence of (detection limit) HBsAg (1 ng/ml), HBsAb (30mIU/ml), HBeAg [2 National Clinical Units (NCU)/ml], HBeAb (2 NCU/ml) and HBcAb (2 NCU/ml) simultaneously in one cassette using a lateral flow OnSiteHBV-5 test, an antibody sandwich immunoassay (CTK Biotech, Inc, USA). The clinical performance characteristics of all the 5 HBV biomarkers are good with relative sensitivities of 100%, and relative specificities ≥ 99.5%.

Women testing positive for just the HBsAg biomarker only in pregnancy were re-tested for other HBV biomarkers seroconversions every 6 months until 24 months.

HBV-exposed babies were tested for HBsAg from birth to 6 weeks of age to determine the vertical HBV transmission rate, and further tested thereafter until 2 years of age to assess any presence of horizontal transmission. Anti-HBs antibody develops during convalescence following acute HBV infection or vaccination. Anti-HBs antibody seroconversion was assessed from 6 months of age until 2 years.

### Data management and statistical analysis

Data were entered and managed using Research Electronic Data Capture (REDCap v 8.0, © 2020). Quality assurance on the accuracy of data entry included independent double entries and verification in cases of discrepancies.

### Statistical analyses

For continuous variables, normalcy testing was performed using the Shapiro–Wilk test. Descriptive summary statistics were calculated for predictor and outcome variables of interest using frequencies and percentages for categorical variables, and medians and interquartile ranges, minimum–maximum for continuous variables. A *p*-value < 0.05 was considered significant.

In a univariate analysis, risk behavior, presence of an STI, common invasive medical procedures of HBsAg sero-positive and HBsAg sero-negative pregnant women were compared using the Kruskal Wallis test, Mann–Whitney U test or Fisher’s exact test where appropriate. Multivariable logistic regression analyses were performed to estimate the odds ratios (OR) and 95% confidence intervals (CI) for factors associated with HBsAg seropositivity. We used automated variable elimination to obtain simplified models with less parameters but high predictive power. Variable elimination was performed for maximizing the Akaike information criterion which considers both the model fit and the number of parameters (i.e. the Akaike information criterion increases with a better model fit but decreases according to the number of predictors) [18]. To test for predictors of HBsAg seropositivity, we carried out multivariate logistic regression analyses both without and with automated variable elimination. In the initial model (without variable elimination), independent variables such as HIV infection, liver enzymes, tooth extractions, blood transfusion and tattooing were included. Statistical analyses were performed in R Studio, version 4.1.1.

### Ethical approval and consent to participate

UZBCS complied with the ethical principles of the Declaration of Helsinki (updated 2013) and was conducted in compliance with the international council for harmonisation of Good Clinical and Laboratory Practice guidelines and local regulatory requirements. Ethical approval was obtained from the Joint Research Ethics Committee (JREC) of the University of Zimbabwe, reference number; JREC/18/15 and the Medical Research Council of Zimbabwe; MRCZ/A/1968.

## Results

### HBsAg seropositivity rate in pregnancy

Overall antenatal HBsAg seropositivity was 32/1208: 2.65%, 95% CI [1.74, 3.55] being; 7/600: 1.17%, 95% CI [0.37, 1.97] versus 25/608: 4.11%, 95% CI [2.52, 5.68] in HIV-uninfected and -infected subgroups respectively. Among the 32 HBsAg seropositive pregnant women 25/32: 78.1%, 95% CI [63.8, 92.4] were HIV-infected compared to 7/32: 21.9%, 95% CI [7.6, 36.2] who were HIV-uninfected, *p* < 0.001. In addition, HBsAg presence divided the women on ethnic lines, with infections disproportionately present in Shona speaking women 27/32: 84.4%, 95% CI [71.8, 96.8] compared to 5/32: 15.6%, 95% CI [3.04, 28.2] in non–Shonas, *p* < 0.001, Table 1. As expected, 31/32: 96.9%, 95% CI [90.8, 100] HBsAg carriers were older, born before 1996, the year the universal hepatitis B vaccine was established in Zimbabwe.

**Table 1:** Socio-demographic, risk behaviour characteristics, clinical data, pregnancy/infant outcomes of pregnant women at least 20 weeks’ gestational age. All = 1208; stratified by maternal HBsAg sero-status into 2 groups: HBsAg positive (*N* = 32) and HBsAg negative (*N* = 1176). Data are expressed as n (%) or median (IQR); min–max unless stated otherwise

Variables Median (1^st^ quartile-3^rd^ quartile); min–max	HBsAg status in pregnancy; *N* = 1208		
**Maternal HBsAg status**	**HBsAg positive: *N* = 32 (2.65%)**	**HBsAg negative: *N* = 1176 (97.35%)**	***p* value**
**Other HBV biomarkers**			
**Anti-HBs-antibodies**			
Positive	0	No further test done	-
Negative	32 (100%)		
**HBeAg**			
Positive	0	No further test done	-
Negative	32 (100%)		
**Anti HBe-antibodies**			
Positive	15 (47.9%)	No further test done	-
Negative	17 (53.1%)		
**Anti-HBc-total antibodies**			
Positive	20 (62.5%)	No further tests done	-
Negative	12 (37.5%)		
**HBsAg only at baseline**			
Yes	10 (31.3%)	No further test done	-
No	22 (68.7%)		
**HIV status, social demography and life style**			
**Maternal HIV Status**			
Positive	25 (78.1%)	583 (49.6%)	**2.2e**^**−16**^
Negative	7 (21.9%)	593 (50.4%)	
**Maternal age (years)** [Median (IQR); min–max]	32 (27.0–35.3); 19–40	28 (23–33); 15–46	**0.012**
**Maternal HBV vaccination in infancy**			
≤ 20 years	1 (3.1%)	84 (7.1%)	0.713
> 20 years	31 (96.9%)	1092 (92.9%)	
**Marital status**			
Not married	2 (6.3%)	55 (4.7%)	**2.2e**^**−16**^
Married	30 (93.8%)	1121 (95.3%)	
**Maternal ethnicity**			
Shona	27 (84.4%)	982 (83.6%)	
Non-Shona	5 (15.6%)	192 (16.4%) (missing = 2)	**2.2e**^**−16**^
**Alcohol use in general (ever)**			
Yes	3 (9.4%)	131 (11.1%)	**2.2e**^**−16**^
No	29 (90.6%)	1045 (88.9%)	
**Alcohol use the index pregnancy**			
Yes	1 (3.1%)	119 (10%)	**2.2e**^**−16**^
No	31 (96.9%)	1057 (90%)	
**Risk Behaviour/procedures**			
**Age at sexual debut** [Median (IQR); min–max]	20 (18–22);14–28	19 (18–21);10–35	0.2902
**Age at first pregnancy** [Median (IQR); min–max]	20.5 (19–24);14–32	20 (18–22);14–40	0.0948
**Presence of casual sex partner(s)**			
Yes	1 (3.1%)	9 (0.8%)	**2.2e**^**−16**^
No	31 (96.9%)	1166 (99.2%) (missing = 1)	
**All your children/pregnancies sharing same father**			
Yes	18 (56.3%)	896 (76.2%)	**2.2e**^**−16**^
No	14 (44.7%)	280 (23.8%)	
**Total number of life sexual partners to date** [Median (IQR); min–max]	2 (1–3);1–30	1 (1–2);1–200	0.0664
**Use of vagina tightening herbs**			
Yes	5 (15.6%)	84 (7.1%)	**2.2e**^**−16**^
No	27 (84.4%)	1092 (92.9%)	
**Any tattoos**			
Yes	2 (6.3%)	86 (7.3%)	**2.2e**^**−16**^
No	30 (93.8%)	1088 (92.7%) (missing = 2)	
**Tooth extraction**			
Yes	11 (34.8%)	203 (17.3%)	**2.2e**^**−16**^
No	21 (65.6%)	973 (82.7%)	
**Blood donation**			
Yes	4 (12.5%)	157 (13.4%)	**2.2e**^**−16**^
No	28 (87.5%)	1019 (86.6%)	
**Blood transfusion**			
Yes	0 (0%)	33 (2.8%)	**2.2e**^**−16**^
No	32 (100%)	1143 (97.2%)	
**STIs in pregnancy**			
**Syphilis antibodies**			
Positive	3 (9.4%)	50 (4.3%)	**2.2e**^**−16**^
Negative	29 (90.6%)	1124 (95.7%) (missing = 2)	
**Abnormal discharge**			
Yes	7 (21.9%)	126 (10.7%)	**2.2e**^**−16**^
No	25 (78.1%)	1050 (89.3%)	
**Itchy vulva**			
Yes	6 (18.8%)	144 (12.3%)	**2.2e**^**−16**^
No	26 (81.2%)	1031 (87.7%) (missing = 1)	
**Genital sores**			
Yes	1 (3.1%)	21 (1.8%)	**2.2e**^**−16**^
No	31 (96.9%)	1154 (98.2%) (missing = 1)	
**Pelvic inflammatory diseases**			
Yes	3 (9.4%)	40 (3.4%)	**2.2e**^**−16**^
No	29 (90.6%)	1136 (96.6%)	
**Presence of genital warts**			
Yes	6 (18.8%)	34 (2.9%)	**2.2e**^**−16**^
No	26 (81.2%)	1138 (97.1%) (missing = 4)	
**Reported previous STI diagnosis**			
Yes	12 (37.5%)	261 (22.2%)	**2.2e**^**−16**^
No	20 (62.5%)	915 (77.8%)	
**Reported previous STI treated (*n* = 273)**			
Yes	8 (66.7%)	172 (65.9%)	**2.2e**^**−16**^
No	4 (33.3%)	89 (34.1%)	
**Clinical data**			
**Aspartate aminotransferase (U/L)** [Median (IQR); min–max]	19.4 (15.5–23.2);2.4–35.0	19.0 (14.0–25.0);0.8–127.0	0.901
**Alanine aminotransferase (U/L)** [Median (IQR); min–max]	10.0 (6.0–14.0);0.3–18.3	11.0 (7.8–15.0);0–76.0	0.168
**Gamma-glutamyl transferase (U/L)** [Median (IQR); min–max]	13.4 (9.5–24.0);2.3–50.9	11.0 (6.70–18.65);0–136.0	0.865
**Alkaline phosphatase (U/L)** [Median (IQR); min–max]	101.3 (72.4–141.3);35.0–277.0	100.4 (74.67–141.0);1.2–865.0	0.245
**Albumin (g/L) categorised**			
< 35	22 (71.0%)	539 (59.2%)	**0.017**
≥ 35	9 (29.0%) (missing = 1)	371 (40.8%) (missing = 66)	
**Total White blood cell count (10^9/L)** [Median (IQR); min–max]	5.8 (4.6–6.9);0.8–10.5	6.8 (5.6–8.1);0.6–42.0	**0.019**
**Haemoglobin (g/dL)** [Median (IQR); min–max]	10.7 (10.3–11.7);8.1–13.5	11.2 (10.2–12.0);1.2–105.0	0.395
**Mid upper arm circumference (cm)** [Median (IQR); min–max]	28.0 (26.0–30.6);24.0–40.0	27.5 (25.0–30.0);20.0–80.0	0.283
**BMI** [Median (IQR); min–max]	25.9 (23.6–29.5);20.3–40.8	25.7 (23.3–28.8);16.3–170.6	0.574
**Previous reported/current TB treatment**			
Yes	2 (6.3%)	57 (4.9%)	**2.2e**^**−16**^
No	30 (93.8%)	1111 (95.1%) (missing = 8)	
**HIV and ART related factors (*n* = 608) **			
**Duration of ART use** [Median (IQR); min–max]	350.0 (87.0–1477.0); 0–3032.0	320.0 (27.0–1256.0); 0–5132.0	0.4208
**CD4**^**+**^** T-lymphocyte count (cells/µL)** [Median (IQR); min–max]	302.5 (233.0–524.5);104.0–730.0	396.0 (258.0–531.0);26.0–1153.0	0.2821
**CD4**^**+**^** T-lymphocyte count (cells/µL)**			
< 350	13 (54.17%)	224 (40.51%)	0.2066
≥ 350	11 (45.83%) (missing = 1)	329 (59.49%) (missing = 30)	
**Viral load (copies/mL)** [Median (IQR); min–max]	0 (0–150);0–15191	0 (0–676);0–614706	0.5084
**Viral load (copies/mL)**			0.8688
≤ 50	17 (68%)	354 (60.7%)	
51–1000	3 (12%)	99 (17.0%)	
1001 -10 000	2 (8%)	40 (6.9%)	
> 10 000	3 (12%)	90 (15.4%)	
**Spouse factors including HIV and ART related factors**			
**Age differences (years)** [Median (IQR); min–max]	4 (2.0–7.3);-9–26.0	5 (3–9);-10–34.0	0.3349
**Living together under 1 roof**			
Yes	30 (93.8%)	1113 (94.6%)	**2.2e**^**−16**^
No	2 (6.3%)	63 (5.4%)	
**Spouse tested for HIV**			
Yes	22 (68.8%)	846 (72.2%)	**2.2e**^**−16**^
No	10 (31.3%)	326 (27.8%) (missing = 4)	
**Pregnancy outcomes (*N* = 1221 infants, including 13 sets of twins) **			
**Maternal HBsAg status**	**Positive: *N *= 33**	**Negative: *N* = 1188**	
			-
**Delivery < 37 weeks gestational age**			
Yes	5 (15.2%)	114 (9.9%)	**0.009**
No	28 (84.8%)	1040 (90.1%) (missing = 34)	
**Birth weight (g)** [Median (IQR); min–max]	3200 (2950–3425); 2100–3990	3100 (2770–3400); 900–4600	**1.22e**^**−05**^
**Birth weight (g)**			
<2500	3 (9.1%)	139 (11.8%)	**0.0004**
>=2500	30 (90.9%)	1034 (88.2%) (missing = 15)	
**Birth length (cm)** [Median (IQR); min–max]	50.0 (48.0–51.0); 45.0–55.0	50.0 (48.0–51.0); 27.0–58.0	0.970
**Infant death over 24 months**			
Yes	0	59 (5%)	**2.2e**^**−16**^
No	33 (100%)	1125 (95%) (missing = 4)	
**MUAC 24 months (cm)** [Median (IQR); min–max]	16.0 (15.0–16.0);13.5–17.0	15.30 (14.50–16.0);10–19	0.540
**HIV vertical transmission by 24 months (*n* = 618, including twins sets)**			
Yes	1 (4.0%)	14 (2.4%)	0.511
No	24 (96.0%)	579 (97.6%)	
**HBV vertical transmission by 24 months of age**			
Yes	0	Not further tested	
No	33 (100%)		-
**Infant Anti-HBs-antibodies from at least 6 months of age**			
Positive	23 (76.6%)	Not further tested	-
Negative	7 (23.3%) (not tested = 3)		

Statistical analysis: comparison of the 2 groups using the Kruskal Wallis test, Mann–Whitney U test or Fisher’s exact test where appropriate. *P*-values in bold font are statistically significant

*Abbreviations ART* Antiretroviral therapy, *BMI* Body mass index, *CD4* Cluster of differentiation 4, *HBV* Hepatitis B, *HIV* Human immunodeficiency virus, *HBeAg* Hepatitis B e antigen, *HBc-antibody* Hepatitis B core antibody, *HBsAg* Hepatitis B surface antigen, *HBs-antibody* Hepatitis B surface antibody, *MUAC* Mid-upper arm circumference, *STIs* Sexually transmitted infections, *WBC* White blood cell count, *IQR* Interquartile range

As expected, none of the 32 HBsAg sero-positive women tested positive for anti-HBs-antibodies. In addition, none of these women were positive for the biomarker of viral replication, HBeAg. Interestingly, 47.9% tested positive for anti-HBe-antibodies, whilst 62.5% were positive for anti-HBc-total antibodies.

Of the 15 women who seroconverted from HBeAg to anti-HBe status, 11 (73.3%) were on ART, Table 1. One woman (6.7%) was HIV-infected but ART naïve (was yet to commence) and three (20%) were HIV-uninfected.

10/32: 31%, 95% CI [15.3, 47.2] were seropositive for HBsAg only with no other HBV biomarkers detected in pregnancy. However, HBs-antibodies seroconversions were later detected in 8/10 (80%) in 6–24 months’ later after delivery. The other 2 women continued testing HBsAg positive without seroconversion or presence of any other HBV biomarker(s) up until 2 years after delivery.

### Comparison between HBsAg-seropositive and HBsAg-seronegatives

In a univariate analysis, HBsAg-seropositive women were older, median age 32 (27–35.3) versus 28 (23–33) years, *p* = 0.012. Furthermore, they were more likely to be married, *p* < 0.001 whilst the frequency of alcohol use, both in general and during pregnancy were significantly higher in the HBsAg negatives both *p values* < 0.001. The ages of sexual debut and at first pregnancy were comparable between the two groups, Table 1.

The frequency of reported presence of casual sexual partners, children/pregnancies not sharing the same father and use of sexual enhancing practices like vagina tightening herbs were significantly higher in HBsAg seropositive women relative to their seronegative peers, *p* values < 0.001 for all the 3 factors, but with comparable medians of total numbers of life time sexual partners, Table 1.

Frequency of reported tooth extraction(s) was higher in HBsAg-seropositive women compared to their HBsAg seronegative counterparts, *p* < 0.001. Interestingly, the latter group reported significantly higher frequency of small skin incision traditional procedures “kutemwa nyora” in vernacular *p* values < 0.001, Table 1. This involves use of razor blades to deliver powdery herbal medication under the skin.

HBsAg seropositive women were significantly more likely to have syphilis antibodies detected, and reported presence of previous STIs, *p* value < 0.001. Of concern were comparable rates of reported previous but untreated STIs in about 34% of women in both groups, Table 1.

Serum albumin levels < 35 g/L were observed in 71% versus 59.2% while lower total white blood counts, median (IQR) 5.8 (4.6–6.9) versus 6.8 (5.6–8.1) were observed in HBsAg seropositive compared to the HBsAg seronegative women, *p* = 0.017 and 0.019, respectively. However, liver enzymes, haemoglobin levels, MUAC and BMI were comparable.

### Comparison between HBsAg/HIV coinfected and HBsAg mono-infected pregnant women

In a univariate analysis comparing 25 HBsAg/HIV coinfected, they were older 32 (29–36) versus 27 (26–31) years versus HBV mono-infected (without HIV infection), *p* = 0.049. Furthermore, they tended to report higher numbers of life time sexual partners to date median (IQR); mini-max of 2 (1–3); 1–30 versus 1 (1–1); 1–1, higher frequency of pregnancies/children not sharing the same father 56% versus 0%, and were more likely to be anemic, haemoglobin levels 10.45 g/dL (9.90–11.53) versus 11.70 g/dL (11.20–11.75), *p* = 0.010, 0.004 and 0.031, respectively. Otherwise, all other maternal factors including pregnancy outcome, infant mortality were comparable between the two groups, Table 2.

**Table 2:** Socio-demographic, risk behaviour characteristics, clinical data, pregnancy/infant outcomes of HBV infected pregnant women at least 20 weeks gestational weeks age (*N* = 32) stratified by HIV status, HBsAg positive/HIV infected (*N* = 25) and HBsAg positive/HIV-uninfected (*N* = 7). Data are expressed as n (%) or median (IQR); min–max, unless stated otherwise

Variables	HBsAg positive pregnant women; N = 32		*p*-value
	**HBsAg-positive/HIV-uninfected pregnant women, *N* = 7 (21.9%)**	**HBsAg-positive/HIV-infected pregnant women, *N* = 25 (78.1%)**	
**Social demography and life style**			
**Maternal age (years)** [Median (IQR); min–max]	27.00 (26.00–31.00); 19.0–32.0	32.00 (29.00–36.00); 21.0–40.0	**0.049**
**Maternal HBV vaccination in infancy**			
≤ 20 years	1 (14.3%)	0	0.219
> 20 years	6 (85.7%)	25 (100%)	
**Difference of spouse/partner age** [Median (IQR); min–max]	4.00 (2.00–5.50); 0–7.0	4.00 (2.00–9.00);-9.0–26.0	0.450
**Marital status**			
Married	6 (85.7%)	24 (96.0%)	0.395
Not married	1 (14.3%)	1 (4.0%)	
**Maternal ethnicity**			
Shona	7 (100%)	20 (80%)	0.560
Non-Shona	0	5 (20%)	
**Alcohol use in general (ever)**			
No	7 (100%)	23 (92.0%)	1.000
Yes	0 (0%)	2 (8.0%)	
**Alcohol use in pregnancy**			
No	7 (100%)	24 (96.0%)	1000
Yes	0 (0%)	1 (4.0%)	
**Risk behaviour/procedures**			
**Age at sexual debut** [Median (IQR); min–max]	21.00 (19.50–22.00);18.0–28.0	19.00 (17.00–22.00);14.0–28.0	0.272
**Age at first pregnancy** [Median (IQR); min–max]	21.00 (19.50–23.00);18.0–28.0	20.00 (19.00–24.00);14.0–32.0	0.784
**All children/pregnancies sharing the same father**			
No	0(0%)	14 (56.0%)	**0.010**
Yes	7 (100%)	11 (44.0%)	
**Total number of sexual partner to date** [Median (IQR); min–max]	1.00 (1.00–1.00);1.0–1.0	2.00 (1.00–3.00);1.0–30.0	**0.004**
**Any Tattoos**			
No	7 (100%)	23 (92.0%)	1.000
Yes	0 (0%)	2 (8.0%)	
**Tooth extraction**			
No	5 (71.4%)	16 (64.0%)	1.000
Yes	2 (28.6%)	9 (36.0%)	
**Blood donation**			
No	7 (100%)	21 (84.0%)	0.552
Yes	0 (0%)	4 (16.0%)	
**Blood transfusion**			
No	7 (100%)	25 (100%)	1.000
Yes	0	0	
**STIs in pregnancy**			
**Syphilis antibodies**			
No	6 (85.7%)	23 (92.0%)	0.536
Yes	1 (14.3%)	2 (8.0%)	
**Abnormal discharge**			
No	7 (100%)	18 (72.0%)	0.300
Yes	0 (0%)	7 (28.0%)	
**Itchy vulva**			
No	6 (85.7%)	20 (80.0%)	1.000
Yes	1 (14.3%)	5 (20.0%)	
**Clinical data**			
**Aspartate aminotransferase (U/L)** [Median (IQR); min–max]	20.00 (17.62–22.00);17.0–28.0	19.40 (12.75–23.40);2.4–35.0	0.568
**Aspartate aminotransferase (U/L) categorised**			
< 10	0	2 (8.3%)	1.000
≥ 10	6 (100%) (missing = 1)	22 (91.7%) (missing = 1)	
**Alanine aminotransferase (U/L)** [Median (IQR); min–max]	10.00 (8.50–11.28);7.0–14.0	10.00 (5.29–14.39);0.3–18.3	0.872
**Alanine aminotransferase (U/L) categorized**			
< 10	2 (33.3%)	11 (47.8%)	0.663
≥ 10	4 (66.7%) (missing = 1)	12 (52.2%) (missing = 2)	
**Total white blood cell count (10^9/L)** [Median (IQR); min–max]	5.80 (5.00–7.30);4.5–8.7	5.80 (4.62–6.80);0.8–10.5	0.825
**Haemoglobin (g/dL)** [Median (IQR); min–max]	11.70 (11.20–11.75);10.6–13.5	10.45 (9.90–11.53);8.1–12.5	**0.031**
**MUAC (cm)** [Median (IQR); min–max]	26.30 (26.00–31.50);24.0–33.0	28.00 (26.00–30.50);24.0–40.0	0.855
**BMI** [Median (IQR); min–max]	25.39 (23.08–30.39);21.6–34.6	25.96 (23.62–29.38);20.3–40.8	0.838
**Pregnancy and infant outcomes (*N* = 33 infants, including 1 sets of twins)**			
	**Infants born to HBsAg-positive, HIV-uninfected women,* N* = 7 (21.2%)**	**Infants born to HBsAg-positive, HIV-infected women, *N* = 26 (78.8%)**	
**Delivery < 37 weeks gestational age**			
No	0	5 (19.2%)	
Yes	7 (100%)	21 (80.8%)	0.559
**Birth weight (grams)** [Median (IQR); min–max]	3400 (3100–3612);2600–3990	3165 (2912–3398);2100–3800	0.252
**Birth length (cm)** [Median (IQR); min–max]	48 (48.00–50.50);47.0–55.0	50 (49–51);45.0–55.0	0.577
**Still births**			
No	7 (100%)	26 (100%)	1.000
Yes	0	0	
**Neonatal deaths (within 28 days)**			
No	7 (100%)	26 (100%)	1.000
Yes	0	0	
**Infant death (after 28 days)**			
No	7 (100%)	26 (100%)	1000
Yes	0	0	

Statistical analysis: comparison of the 2 groups using the Kruskal Wallis test, Mann–Whitney U test or Fisher’s exact test where appropriate. *P*-values in bold font are statistically significant

*Abbreviations BMI* Body mass index, *HBV* Hepatitis b virus, *HIV* Human immunodeficiency virus, *IQR* Interquartile range, *MUAC* Mid upper arm circumference, *STIs* Sexually transmitted infections

Of the 25 HBsAg/HIV coinfected 5/25: 20%, 95% CI [4.32, 35.68] were virally unsuppressed (HIV-RNA > 1000 copies/mL) with 13/25: 54.4%, 95% CI [25.7, 78.3] being immune compromised, with CD4 + T-lymphocyte counts < 350 cells/µL, not shown.

Due to the higher HIV seropositivity rates in HBsAg seropositive women the higher frequency of previous/current tuberculosis infection observed was not surprising compared to the HBsAg mono-infected, *p* < 0.001, Table 1.

Frequency of other HBV serological biomarkers; anti-HBsAb, HBeAg, anti-HBe, anti-HB-core-antibodies biomarkers, were comparable between the HBV-mono-infected and HBV/HBV co-infected pregnant women, not shown.

### HBsAg seropositivity and adverse birth outcomes and mortality rates

Five of the 33 HBV-exposed babies (including 1 set of twins) were born prematurely (< 37 weeks) 15.2% versus 9.9% in HBsAg seropositive women compared to the HBsAg seronegatives, respectively *p* = 0.009, Table 1. All the 5 premature births were from the HBsAg positive/HIV-infected group, and of these women, 4 had HIV RNA load < 1000 copies/mL.

Median birth weight was significantly higher, median (IQR) 3200 g (2950–3425) versus 3100 g (2770–3400) in HBsAg seropositive women compared to seronegatives, *p* < 0.001. Interestingly, no mortality was observed in the former group compared to the 5% mortality rate in the latter. The positive impact of ART is not surprising as HBsAg seropositive women were on therapy for almost a year, median IQR 350 (87–1477) days of ART use, and 80% had HIV-RNA < 1000 copies per mL, Table 1.

Birth length, infant MUAC, including HIV-MTCT rates were comparable between the HBsAg-exposed and unexposed children over the 24 months’ follow-up period, Table 1.

### HBV-MTCT rate and infant HBs-antibody seroconversion following vaccination

None of the HBV-exposed infants tested HBsAg positive from birth up until 2 years of age. Thus, both the vertical and horizontal HBV-MTCT rate were 0%. In HBV exposed infants with documented records for evidence of receipt of all the 3 doses of HBV vaccine in the Road to Heath Baby Cards, anti HBs-antibodies were detected in 23/30: 76.6%, 95% CI [55.17, 98.17] (3 babies were not tested). Thus, no HBs-antibodies were detected at 2 years of age in 23.3% of HBsAg-exposed infants, despite having successfully completed the HBV vaccination schedule.

There were no significant differences in maternal factors such as HIV status, MUAC including infant factors; sex, prematurity birth weight analysis between babies who had anti-HBs-antibodies present (3 babies were not tested) compared to those without anti HBs-antibodies being detected; Table 3.

**Table 3:** Factors associated with immunoprophylaxis success or failure in infants born of HBsAg seropositive women tested for HBs antibodies from at least 6 months of age

Variables	HBsAg positive pregnant women whose infants were tested for HBsAb at minimum 6 months of age; (*N* = 29)		*p*-value
	**Immunoprophylaxis success (*n *= 22)**	**Immunoprophylaxis failure (*n* = 7)**	
**Maternal factors**			
**Maternal age (years)** [Median (IQR); min–max]	31 (27–35.25); 21–40	32 (27.50–35); 19–36	0.878
**Maternal BMI** [Median (IQR); min–max]	25.51 (23.04–28.25); 20.70–40.77	27.86 (24.50–33.59); 20.32–39.74	0.415
**Maternal MUAC** [Median (IQR); min–max]	27.50 (25.25–30.0); 24–38	28 (26.65–34.50); 26–40	0.209
**Maternal HIV Status**			
Positive	16 (72.7%)	6 (85.7%)	0.728
Negative	6 (27.3%)	1 (14.3%)	
**Duration of ART use** [Median (IQR); min–max]	296 (39–1291.5); 0–3032	1460.5 (594.8–1495.2); 87–1671	0.302
**CD4**^**+**^** T-lymphocyte count (cells/µL)** [Median (IQR); min–max]	352.5 (233–524.5); 104–730	443 (291–567); 169–669	0.509
**CD4**^**+**^** T-lymphocyte count (cells/µL)**			
< 350	8 (50%)	2 (40%)	1.000
≥ 350	8 (50%)	3 (60%) (missing = 1)	
**Viral load (copies/mL)** [Median (IQR); min–max]	19 (0–170); 0–15191	0 (0–0); 0–19	0.076
**Viral load (copies/mL) categorised**			
≤ 50	10 (62.5%)	6 (100%)	
51–1000	3 (18.8%)	0	0.561
1001 -10 000	1 (6.2%)	0	
> 10 000	2 (12.5%)	0	
**Infant factors (*n* = 30)**			
	**Presence of HBs-antibodies at 2 years (*n* = 23, including 1 twin set)**	**Presence of HBs-antibodies at 2 years (*n* = 7)**	
**Infant sex**			
Male	11 (47.8%)	4 (57.1%)	1.000
Female	12 (52.2%)	3 (42.9%)	
**Delivery < 37 weeks gestational age**			
No	19 (82.6%)	6 (85.7%)	1.000
Yes	4 (17.4%)	1 (14.3%)	
**Birth weight (g)**			
<2500	2 (8.7%)	1 (14.3%)	1.000
>/= 2500	21 (91.3%)	6 (85.7%)	

Statistical analysis: comparison of the 2 groups using the Kruskal Wallis test, Mann–Whitney U test or Fisher’s exact test where appropriate. *P*-values in bold font are statistically significant

*Abbreviations ART* Antiretroviral therapy, *BMI* Body mass index, *CD4* Cluster of differentiation 4, *HIV* Human immunodeficiency virus, *IQR* Interquartile range, *MUAC* Mid-upper arm circumference

### Predictors of antenatal HBsAg seropositivity

In a multivariate logistic regression model, with variable elimination, we observed significant associations of a positive maternal HIV status and reported tooth extraction(s) with HBsAg-seropositive, odds ratio (CI): 3.85 (1.61–10.7) and 2.46 (1.07–5.34), respectively, Table 4.

**Table 4:** Predictors of HBsAg sero-positivity. Predictors of HBsAg sero-positivity in pregnant women of at least 20 weeks of gestational age without variable elimination (423 observations) and with variable elimination (514 observations)

	Predictors of HBsAg sero-positivity					
	Without variable elimination			With variable elimination		
	OR	CI	*p*-value	OR	CI	*p*-value
Intercept	1.51e^−03^	7.11e^−05^- 3.28 e^−02^	1.39e^−05^	0.01	0.004 – 0.04	2.07e^−14^
Maternal HIV status (Positive)	3.48	1.41–9.95	**0.011**	3.85	1.61 – 10.70	**0.005**
Tooth extraction (Yes)	2.09	0.88 – 4.73	0.083	2.46	1.07 – 5.34	**0.026**
Aspartate aminotransferase** (**per unit)	0.97	0.93 – 1.01	0.201	0.97	0.93 – 1.01	0.194
Gamma-glutamyl transferase (per unit)	1.00	0.98 – 1.02	0.613	1.01	0.98 – 1.03	0.515
Age (per year)	1.04	0.97—1.11	0.253	-	-	-
Casual sex partner (Yes)	2.22	0.11—16.69	0.498	-	-	-
Any tattoos (Yes)	0.79	0.12 – 2.81	0.761	-	-	-
Blood transfusion (Yes)	2.81 e^−07^	2.62e^−165^ – 4.01e^+16^	0.989	-	-	-
Reported STI (Yes)	1.57	0.67 – 3.45	0.272	-	-	-
Albumin (per unit)	1.00	0.95 – 1.03	0.586	-	-	-
Spouse tested HIV (Yes)	1.41	0.61 – 3.75	0.449	-	-	-
Baby weight (> 2500 g)	1.88	0.53 – 11.96	0.404	-	-	-

Statistical analysis: Logistic regression with and without variable elimination

*Abbreviations CI* 95% confidence interval, *HIV* Human immunodeficiency virus, *HBsAg* Hepatitis b surface antigen, *OR* Odds ratio

## Discussion

We report results on HBsAg seropositivity rates and associated risk factors in 608 HIV-infected pregnant on Tenofovir/Lamivudine/Efavirenz regimen with a median of 350 days since ART initiation alongside their 600 HIV-uninfected counterparts the UZBCS.

Maternally HBV-exposed babies were tested for HBsAg from birth, 6 weeks until 2 years. Presence of infants’ HBs-antibodies was evaluated from 6 months of age.

The following were key findings:

- In the study population of 1208 pregnant women, the overall HBsAg sero-prevalence was 2.65%, 95% CI [1.74, 3.55], being almost four times higher in the HIV-infected women 4.11%, 95% CI [2.52, 5.68] against 1.17%, 95% CI [0.37, 1.97] in their HIV-uninfected peers.
- The most important predictors for HBsAg seropositivity were HIV-infection status and reported tooth extraction(s), and these were associated with 3.85 and 2.46 times risks of HBsAg-seropositivity, respectively.
- The frequency of preterm births was significantly higher in HBsAg/HIV positive women.
- HBV-MTCT and horizontal transmission rates in maternally HBV-exposed babies receiving their first dose of HBV-vaccine at 6 weeks of age were both 0%.
- No anti-HBs-antibodies were detected at 2 years of age in 23.3% of fully vaccinated babies.

### Overall HBsAg seropositivity in pregnancy

The overall prevalence of HBV was 2.65% in pregnancy; being 1.17% versus 4.1% in the HIV-uninfected and infected. The overall HBsAg sero-prevalence rate of 2.65% was within the intermediate prevalence category. Nevertheless, this figure was much lower compared to the high prevalence category of > 8% observed during the pre-ART era. This was a further drastic decline from very high prevalence rates reported in previous Zimbabwean studies done in the late 1990s where HBsAg seropositivity rate in pregnant women was 25% [13].

However, the HIV-uninfected subgroup’ HBsAg endemicity fell within the low prevalence category.

Our results are comparable to some regional findings, but not others. HBsAg seroprevalence rates in Cameroonian, Nigerian, Rwandan and Mozambican pregnant women ranged from 3.7%-18.4% [19–22]. Similarly, all these studies were also done during this option B + era of life long ART use_**.**_ The overall prevalence rate of 2.65% observed in our study is the lowest of them all. This is evidence that Zimbabwe is working steadily towards the target 3.3 of the 2016 Sustainable Development Goals aiming to eliminate viral hepatitis as a public health problem by 2030 through optimising strategies for prevention, diagnosis and treatment of HBV infections [2].

In our study, HIV-positive status and reported tooth extraction(s) were independent risk factors for maternal HBsAg seropositivity. However, being born before the universal implementation of routine hepatitis B immunisation and CD4 count < 350 cells/mL predicted HBsAg positivity in a Sierra Leone study [23]. Bigger studies are warranted to further validate and understand modifiable risk factors around reported tooth extractions processes in our setting.

Previous studies have shown that considerable racial/ethnic disparities exist in HBV infection, exposure and immunity [24]. In our study, the Shona speaking women were more likely to test HBsAg positive compared to non-Shonas.

Encouragingly, our study findings were suggestive of safe traditional medical practices that use sharps. In addition, our blood and blood products from the national blood bank seem safe too.

The overall regional HBV-seroprevalence for SSA’s general population of 2.15% [25] is much higher compared to the 1.17% observed in our cohort among HIV-uninfected pregnant women, a figure well below the 2% threshold, meeting the low HBV prevalence classification. The WHO recommends HBV DNA testing and Tenofovir treatment from weeks 28 to 32 of pregnancy for HBV mono-infected women. However, securing access to the more sensitive HBV-DNA testing and the procurement of single dose Tenofovir for the mono-infected remain essential. There is need for HBsAg, testing, monitoring and treatment services in the public sector in the context of continuum of care and in accordance with universal health coverage. For affordability and more practical purposes, this could be done in synergy and coordination with the successful ongoing HIV prevention and control services to preclude new HBV infections.

### HBsAg positive/HIV coinfection

In a Mozambican study, 36.3% of HBsAg-positive pregnant women were HIV coinfected [20]**.** This figure is much lower compared to the 78.1% observed in our cohort study. In our study, the HIV-infected pregnant women were 3.9 times more likely to be infected with HBV compared to their HIV-uninfected peers.

The HBsAg-positive/HIV coinfection rate of 4.11% observed in our study was very similar to the Rwandan seropositivity rate of 4.1% observed in a much bigger cohort of 13,121 pregnant women [19]. Interestingly, both studies were done during this era of life long ART. Both rates are much lower compared to the 12% rate observed in ART naïve Zimbabwean adults [26]. However, the rate of 4.11% observed in our study done in an urban setting was 5 times higher compared to the 0.8% observed in rural pregnant women in 2010 [14]. Probably the risks are lower for the rural peers. However, our observed rate of 4.11% was higher than the overall pooled prevalence of HBV/HIV co-infection among pregnant mothers in SSA which is 3.30% [25].

HBsAg seropositivity has been significantly associated with anemia and elevated ALT levels [27–29] at least in ART naïve adults. However, in our study no significant differences were observed in ALT levels between HBsAg seropositives and negatives. This is not surprising since previous studies demonstrated that serum ALT levels decreased following ART initiation in HIV-infected individuals [30].

ART exposures inhibit HBV replication, and hence could be the explanation underlying the gradual decline of HBV prevalence from as high as 6.36% [25] in reports published during the intrapartum single dose Nevirapine era done between years 2004–2010. The highest prevalence of up to 25% recorded during the pre-ART era in the 1990s [13]. Thus, our conclusion of the current lower HBV burden may not necessarily represent the natural results of transmission due to lifelong Tenofovir/Lamivudine/Efavirenz exposures for HIV therapy. In our study 78.1% of the HBV seropositive women were also coinfected with HIV and were on ART based regimen that also interrupt HBV replication. Tenofovir, which is included in the treatment combinations as first-line therapy for HIV infection, is also active against HBV [11].

### HBsAg seropositivity and adverse birth/infant outcomes

There has been conflicting findings on the association of maternal HBV infection and adverse birth outcomes, especially on preterm deliveries [31]. Our study demonstrated that HIV/HBV-exposed babies tended to be born prematurely, but infant birth lengths were comparable. Interestingly, the maternally HIV exposed had heavier birth weights relative to their HBV-unexposed peers. This could be attributed to the positive impact of successful HIV therapy. About 80% HBsAg/HIV co-infected women were on ART for almost at least one year, hence generally were virally suppressed with a fair median CD4 counts of 305 cells/µL. This could also have been the reason behind comparable HBV-MTCT rates observed in HIV-infected/HBsAg seronegative and HIV infected/HBsAg seropositive women.

### HBV-vertical and horizontal transmission rates

HBV infection in newborns is defined as HBsAg positivity within 6 months after birth. None of our babies were HBV-infected by 6 weeks of age even thereafter until 2 years in our cohort of babies receiving their first HBV vaccine at 6 weeks of age. The detection limits for the HBsAg screening, confirmatory and the third exploratory test were quite low in the 1 ng/mL ranges. Encouragingly all the three test kits had 100% concordance rates, and excellent test performances with respect to sensitivities and specificities.

This low transmission rate is comparable to only one case out of the 134 (0.7%) seropositive of maternally HBV-exposed Mozambican infants who received their first HBV vaccine at birth [20]. Previous studies have shown that delayed HBV vaccine birth dose is still effective in preventing perinatal infection, although at a reduced efficacy. This is even more important in our study setting where the third HBV vaccination dosage coverage is at least 83%, making our results less generalisable.

HBeAg (detected in serum of individuals with high HBV viral load), anti-HBe-antibodies and anti-HBc-antibodies (IgM/IgG total antibody) indicate recent or past infection. Anti-HBe antibody detection is generally associated with the disappearance of HBeAg, and indicates low infectivity. In our study, up to 47.9% of the pregnant women tested positive for anti-HBe-antibodies compared to only 3.3% observed in the late 1990s. This is critical, as lack of maternal anti-HBe antibodies predicts HBV infection in babies.

Ordinarily, the presence of HBsAg and/or HBV-DNA in neonates is the criteria for determining intrauterine, including perinatal transmissions that can occur before the administration of HBV vaccine at 6 weeks of age. Presence of HBsAg, HBeAg and anti-HBc IgM–antibodies from at least 6 months of age are potentially useful for simple and cost-effective algorithms for early HBV infant diagnosis in resource limited settings. HBV-MTCT rates vary with maternal HBeAg/anti-HBe status, being 70%-90% in HBeAg seropositives, 25% in HBeAg-seronegative/HBeAb negatives and 12% in HBeAg-negative/anti-HBe-positives [32–34]. Approximately 3%-13% of babies born of HBsAg-positive mothers, especially those carrying HBeAg become HBsAg carriers despite successful passive-active immunoprophylaxis [32, 33].

Perinatal HBV diagnosis remains a challenge as even more sensitive and reliable diagnostic platforms have shown that in some cases neonates born of HBV infected pregnant women test HBsAg positive at birth but with absence of infection simply because the small molecular HBsAg passes through the placenta. In addition, anti-HBe antibody and anti-(HBc antibody) also cross the placental barrier in nearly all babies before disappearing before 12 and 24 months, respectively [35, 36]. Again, this is just transplacental maternal antibodies transfers, but not indicators of infant HBV infection status.

Real-time polymerase chain reaction with two pairs of primers and probes has been shown to prevent the underestimation of HBV DNA levels [37], and can also detect occult HBV infection (i.e. HBsAg seronegative individuals but with detectable HBV DNA). Combining HBsAg and HBV DNA assays has been shown to increase prevalence of HBV from 4.8% (HBsAg alone) to 12.4% [38, 39]. Molecular diagnostic inadequacies may lead to significant underestimation of HBV infections. Furthermore, viral mutations, or genetic diversity that differ by geographic location may also contribute significantly to the occult HBV phenotype causing immune escape and diagnostic failures, vaccine escapes [40–45].

### Infant immune response to HBV vaccination

Of concern were the 23.3% of HBV exposed infants that lacked HBs-antibodies at 2 years after receiving all the recommended 3 vaccine doses. Three exposed babies were not tested at 24 months because they had relocated to the rural areas due to economic hardships, a situation exacerbated by the covid-19 pandemic lockdowns. Previous findings suggested that age, obesity and gender differences may affect immune responses to HBV vaccine [46–48]. However, we did not observe these associations in our study, probably due to the small numbers of maternally HBV exposed babies. Host genetic variants may have played a role in this hypo-immune response to HBV vaccine as previously described [49–51]. Being non-cytopathic, liver injury following HBV infection is mostly due to host immune responses [52]. However, no risk factors associated with lack of HBs-antibodies seroconversion were observed, probably because again of the small numbers. This observation resonates well with previously studies’ findings that demonstrated that despite complete HBV vaccinations, a significant number of South African children were not immune to HBV [53]. Policy makers may have to consider revaccination for failed active immunoprophylaxis targeting HBV exposed babies. In addition, and more importantly it remains to be ascertained whether the infants with HBs-antibodies seroconversion had protective antibody levels of at least 10 IU/mL. Further investigations are necessary to ascertain whether lack of antibodies implies lack of immunity.

Birth dose of hepatitis B vaccine is currently not part of the immunization schedule in Zimbabwe, despite the fact that high proportion of deliveries occur within health care facilities [12], a situation that would facilitate its implementation. The current economic hardships now associated with increasing numbers of home births may be a challenge for HBV birth dose implementation if the situation continues. Paradoxically, are recent European studies that recorded perinatal HBV infections of 1% in infants receiving HBV birth dose [54, 55]. Given the foregoing, it may be worthwhile continuing with the current strategy of administering first HBV vaccine at 6 weeks of age.

## Conclusion

In conclusion, in our cohort of 1208 pregnant women in a low-resource setting in SSA, the overall HBsAg sero-prevalence rate was 2.65% in women ≥ 20 weeks’ gestational age. The burden significantly different by HIV status; being 1.17% versus and 4.11% in the HIV-uninfected and infected subgroups, respectively. Thus, low and moderate HBV endemics were observed in HIV-uninfected and HIV-infected pregnant women, respectively, underscoring the need for routine HBV screening in pregnancy, especially the HIV-infected women attending antenatal care. Unlike for HIV, there is no population-based testing for HBV despite the fact that the two viruses share the same modes of transmission. There is no data on national HBV prevalence to help the readers to interpret the low and moderate HBV endemicity seen in our study in the background of the bigger national picture. HIV-infection and reported tooth extraction(s) were independent risk factors for HBsAg-seropositivity, warranting augmentation of respective preventive measures. Concerted efforts are necessary to optimise healthy pregnancy outcomes in the HIV/HBsAg coinfected for a greater impetus towards attaining the WHO strategy aiming to end the HBV epidemic by 2030. Vertical and child-horizontal transmissions were both absent, probably due to ~ 50% frequency of antenatal anti-HBe-antibodies. Of concern was the absence of anti-HBs-antibodies in about a quarter of the fully vaccinated/maternally HBsAg exposed infants. Finally, lack of adequate molecular diagnostic facilities in many African centres may lead to significant underestimation of HBV infections such that both vertical and horizontal transmissions are underappreciated, and hence may not be diagnosed until later in young adulthood.

## Data Availability

The datasets obtained during this study will be available upon reasonable request to the corresponding author.

## Abbreviations

ART: Antiretroviral therapy
BMI: Body mass index
CD4: Cluster of differentiation-4
CI: Confidence interval
DNA: Deoxyribonucleic acid
HBV: Hepatitis B virus
HBc-antibody: Hepatitis B virus core antibody
HIV: Human immunodeficiency virus
HBeAg: Hepatitis B virus e antigen
HBsAg: Hepatitis B virus surface antigen
HBs-antibody: Hepatitis B virus surface antibody
IQR: Interquartile range
MUAC: Mid-upper arm circumference
MTCT: Mother to child transmission
STIs: Sexually transmitted infections
UZBCS: University of Zimbabwe Birth Cohort Study.
WHO: World Health Organisation
